# Hormone Replacement Therapy Reverses Gut Microbiome and Serum Metabolome Alterations in Premature Ovarian Insufficiency

**DOI:** 10.3389/fendo.2021.794496

**Published:** 2021-12-23

**Authors:** Lingling Jiang, Haiyi Fei, Jinfei Tong, Jiena Zhou, Jiajuan Zhu, Xiaoying Jin, Zhan Shi, Yan Zhou, Xudong Ma, Hailan Yu, Jianhua Yang, Songying Zhang

**Affiliations:** ^1^ Assisted Reproduction Unit, Department of Obstetrics and Gynecology, Sir Run Run Shaw Hospital, Zhejiang University School of Medicine, Hangzhou, China; ^2^ Department of Obstetrics and Gynecology, Key Laboratory of Reproductive Dysfunction, Hangzhou, China; ^3^ Department of Obstetrics and Gynecology, Yaojiang Township Central Hospital, Zhuji City, China; ^4^ Department of Medical, Jiaxing University Affiliated Women and Children Hospital, Jiaxing, China

**Keywords:** premature ovarian insufficiency, hormone replacement therapy, untargeted metabolomics, gut microbiota, TGF-β1

## Abstract

**Objective:**

We explored the gut microbiome and serum metabolome alterations in patients with premature ovarian insufficiency (POI) and the effects of hormone replacement therapy (HRT) with the aim to unravel the pathological mechanism underlying POI.

**Methods:**

Fecal and serum samples obtained from healthy females (HC, n = 10) and patients with POI treated with (n = 10) or without (n = 10) HRT were analyzed using 16S rRNA gene sequencing and untargeted metabolomics analysis, respectively. Peripheral blood samples were collected to detect serum hormone and cytokine levels. Spearman’s rank correlation was used to evaluate correlations between sex hormones and cytokines and between the gut microbiota and serum metabolites. To further confirm the correlation between *Eggerthella* and ovarian fibrosis, the mice were inoculated with *Eggerthella lenta* (*E. lenta*) through oral gavage.

**Results:**

The abundance of genus *Eggerthella* significantly increased in the fecal samples of patients with POI compared to that observed in the samples of HCs. This increase was reversed in patients with POI treated with HRT. Patients with POI showed significantly altered serum metabolic signatures and increased serum TGF-β1 levels; this increase was reversed by HRT. The abundance of *Eggerthella* was positively correlated with altered metabolic signatures, which were, in turn, positively correlated with serum TGF-β1 levels in all subjects. Estrogen ameliorated ovarian fibrosis induced by *E. lenta* in mice.

**Conclusions:**

The interactions between the gut microbiota, serum metabolites, and serum TGF-β1 in patients with POI may play a critical role in the development of POI. HRT not only closely mimicked normal ovarian hormone production in patients with POI but also attenuated gut microbiota dysbiosis and imbalance in the levels of serum metabolites and TGF-β1, which are reportedly associated with fibrosis. The findings of this study may pave the way for the development of preventive and curative therapies for patients with POI.

**Graphical Abstract:**
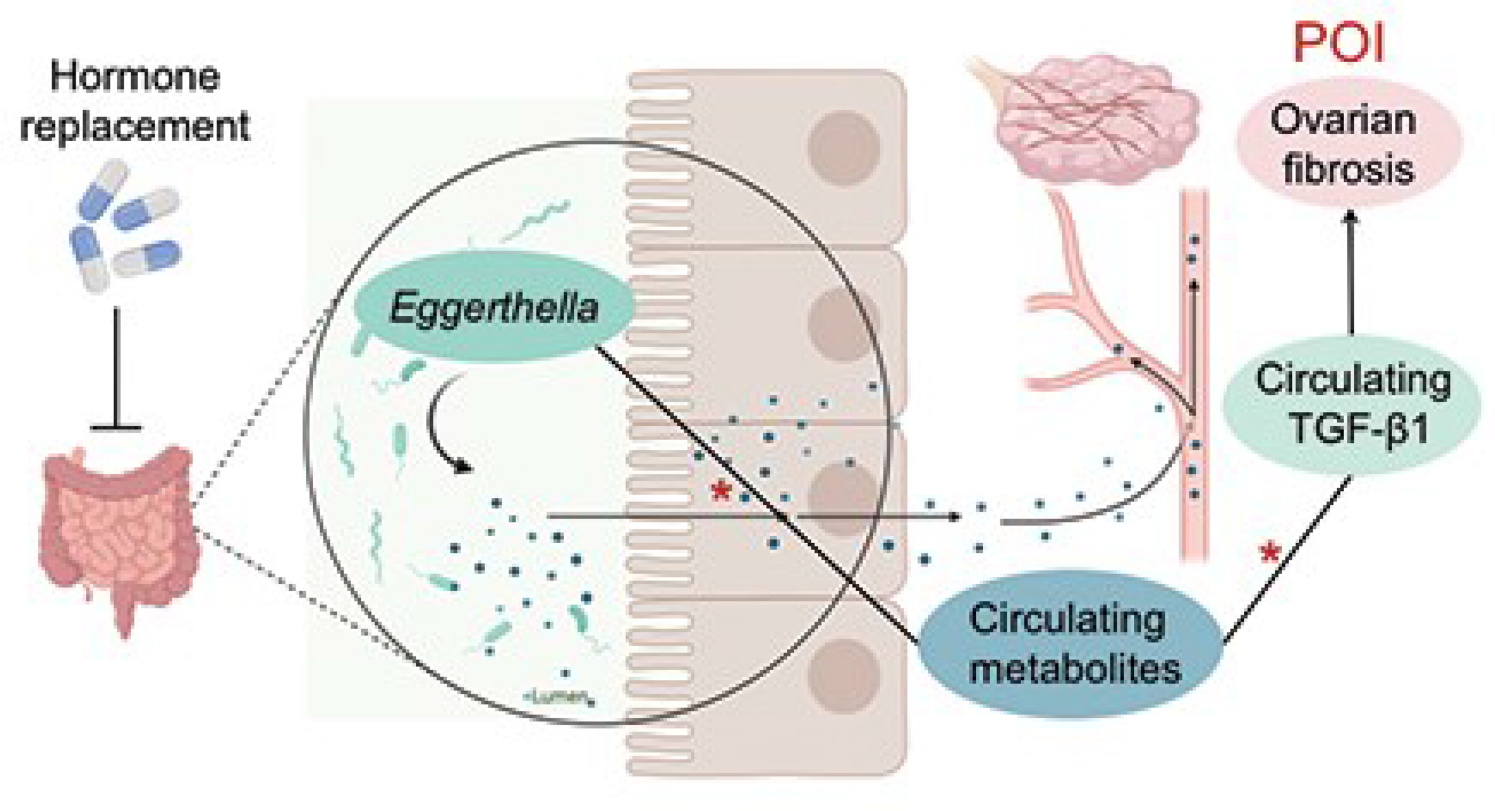
Jiang et al. report that the abundance of Eggerthella significantly increased in fecal samples of patients with premature ovarian insufficiency. Moreover, circulating metabolites and TGF-β1 levels were elevated in these patients. These alterations were all reversed by HRT. The abundance of Eggerthella was positively correlated with the increased circulating metabolites, which were, in turn, positively correlated with the serum levels of TGF-β1, a profibrotic factor. * indicates positive correlations, *P<0.05.

## 1 Introduction

Premature ovarian insufficiency (POI) refers to ovarian functional decline before 40 years of age characterized by abnormal menstruation (amenorrhea or too frequent menses), elevated levels of follicle-stimulating hormone (FSH) (>25 U/L), and low serum levels of estradiol (E2) (1). POI has a variety of etiologies, including genetic, autoimmune, iatrogenic, surgical, and spontaneous manifestations associated with chemotherapy or radiotherapy. The underlying cause of POI in approximately 90% of patients with 46, XX normal karyotype who are younger than 40 years remains unelucidated (2).

Several studies have reported alterations in the gut microbiota of patients with ovarian dysfunction, especially in patients with polycystic ovary syndrome (PCOS) (3, 4). The gut microbiota may also affect metabolite levels in various metabolic pathways in patients with PCOS, especially in lipid metabolism, which, in turn, may further aggravate the imbalance of the intestinal microbiota (5). However, only one recent report identified altered microbial profiles in the gut microbiome of patients with POI. A reduction in the abundance of Phylum *Firmicutes*, genera *Bulleidia* and *Faecalibacterium* and increased abundance of phylum *Bacteroidetes*, genera *Butyricimonas*, *Dorea*, *Lachnobacterium*, and *Sutterella* was observed in females with POI compared to that in healthy controls (6). Therefore, the relationship between POI and the intestinal microbiota remains to be examined.

A recent meta-analysis revealed a correlation between POI and increased risk of type 2 diabetes mellitus (T2DM) (7). Accumulating evidence suggests that the gut microbiota is involved in the pathological development of insulin resistance, and therefore, contributes to the development of type 2 diabetes mellitus by affecting the levels of important metabolites (8, 9). In addition, a recent study revealed that the abundance of multiple metabolites, especially lipids, glycerophospholipids, steroids, and amino acids, was altered remarkably in ovarian tissues after the development of POI in mice and was reversed by the injection of the human umbilical cord mesenchymal stem cells (10). However, whether changes in the gut microbiota profile of patients with POI would lead to an imbalance in serum metabolites and subsequently induce menopausal symptoms and other health risks remains to be elucidated.

Further, the estrogen-gut microbiome axis has a critical impact on the overall health of menopausal females in animal models and humans (11, 12). Intestinal epithelial estrogen receptor beta impacts the gut microbiota diversity in mice (13). Ovariectomy and E2 administration significantly changed the composition of gut microbiota and the abundance of microbial short-chain fatty acids, thereby impacting the overall health in a menopausal rat model (14). A randomized controlled study revealed that hormone replacement therapy (HRT) also significantly reduced the amount of atherogenic lipids in postmenopausal females (15). In fact, appropriate HRT, which replaces premenopausal ovarian sex hormone levels, is essential to improve the quality of life and lower associated health risks in patients with POI. HRT can reduce menopausal symptoms and fracture risk, protect against the early progression of cardiovascular diseases, and improve the emotional health and cognitive function of patients (2). Therefore, we hypothesized that HRT may exert therapeutic effects by altering the gut microbiota and circulating metabolite profiles.

**Figure 1:**
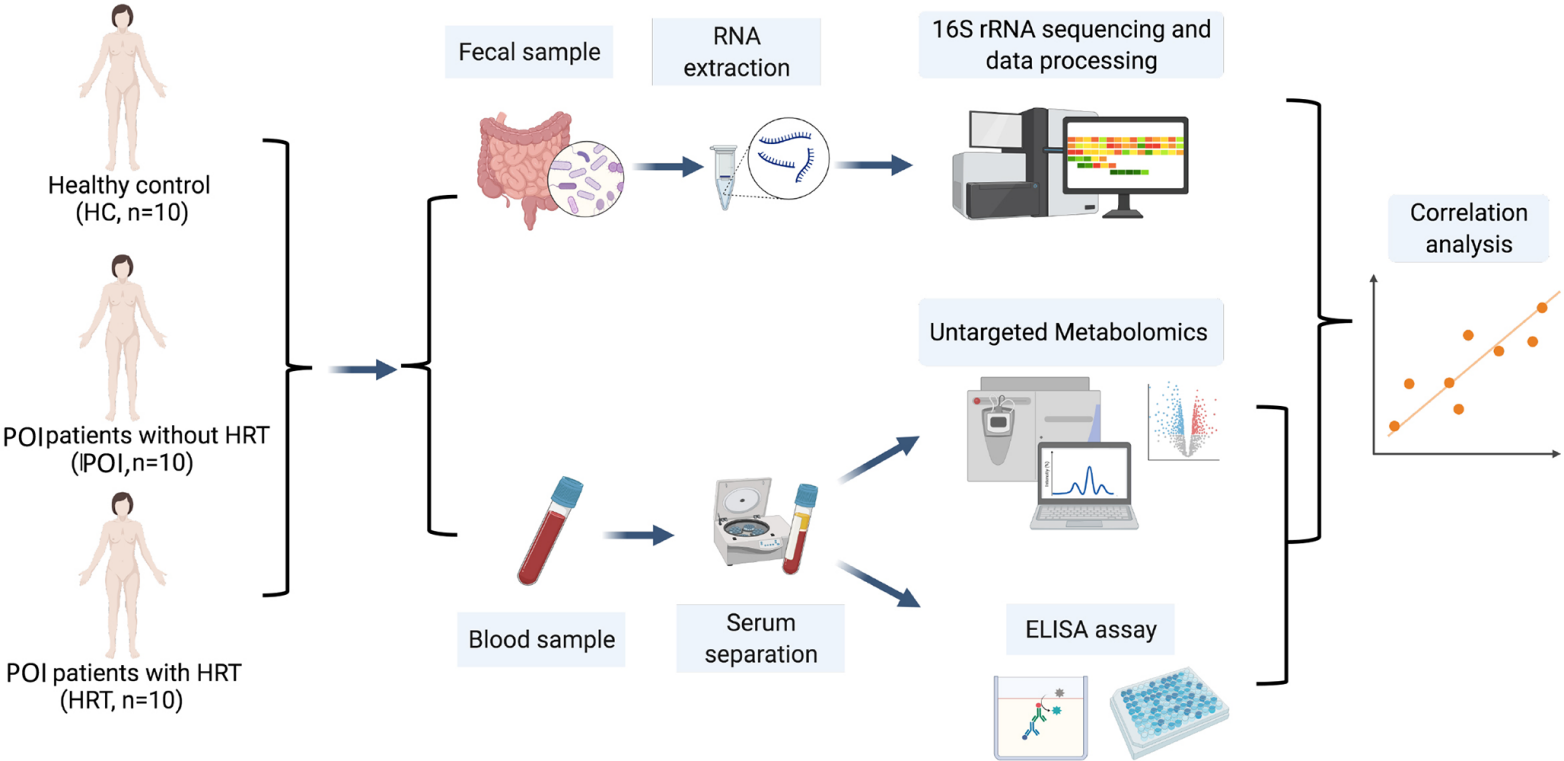
Schematic diagram of the experimental protocol involved in the collection of blood and fecal samples from the participants.

This study aimed to examine the alterations in the intestinal microbiota and serum metabolites in patients with POI and the mechanisms underlying improvements mediated in the menopausal symptoms and other health risks associated with POI by HRT in patients with POI with (n = 10) and without (n = 10) HRT by comparison with healthy females (HCs, n = 10). We aimed to conduct a 16S rRNA gene sequencing analysis of fecal samples and untargeted metabolomics analysis of serum samples and analyze the correlations between the gut microbiota and serum metabolites and among serum metabolites, serum sex hormones, and cytokines (**Figure 1**).

## 2 Materials and Methods

### 2.1 Study Subjects

All participants (HCs, n = 10; patients with POI, n = 10; patients with POI treated with HRT, n = 10) were recruited at the Department of Obstetrics and Gynecology of Sir Run Run Shaw Hospital, Zhejiang University School of Medicine. All subjects provided written informed consent for sample collection and analyses. The study and all experimental procedures were approved by the ethics committee of Sir Run Run Shaw Hospital, Zhejiang University School of Medicine (approved code: keyan20211108-32).

Patients with POI were diagnosed based on the following criteria: oligo/amenorrhea for at least 4 months, and an elevated serum FSH level > 25 IU/L detected at two intervals more than four weeks apart before the age of 40 years (16). All Patients with POI had 46, XX karyotype, not attributed to known etiology, such as genetic abnormalities, autoimmune diseases, pelvic surgery, and chemo/radiotherapy treatment. The patients had no family history of POI (17). The healthy control donors were age-matched females (<40 years) who had regular menstrual cycles, normal levels of FSH (<10 IU/L), and normal levels of estrogen and progesterone. The exclusion criteria included any infected diseases, malignant tumors, intestinal diseases, obesity or other metabolism-related diseases, drug or alcohol use, and antibiotics, probiotics, or prebiotics use in the past three months. All participants in the HRT group received periodic sequential regimens and were treated for more than one year. During the first 11 days of the cycle, 17β-estradiol (2 mg) was administrated orally daily, followed by 7 days of withdrawal, and dydrogesterone (10 mg) was administered daily for 10 consecutive days. In addition, the height and bodyweight of the participants were measured, and the body mass index (kg m^−2^) was calculated.

### 2.2 16S rRNA Gene Sequencing

Genomic DNA extraction and library construction were performed at Shenzhen BGI Technology following a previously reported protocol (18).

#### 2.2.1 Genomic DNA Extraction

Briefly, fresh fecal samples were collected in the morning before breakfast from each participant within one week after the end of menstruation. For participants in menopause, no time limit was set for collection. The middle section of the formed feces was excavated with a disposable sterile feces collector. The samples were rapidly frozen with liquid nitrogen for 5 min and stored at -80°C until microbial DNA extraction. The microbial community DNA was extracted using MagPure Stool DNA KF kit B (Magen, China) following the manufacturer’s instructions.

#### 2.2.2 Library Construction

The variable regions V4 of bacterial 16S rRNA gene were amplified using degenerate PCR primers, 515F (5′-GTGCCAGCMGCCGCGGTAA-3′) and 806R (5′-GGACTACHVGGGTWTCTAAT-3′), and the PCR cycling conditions were set as follows: 95°C for 3 min, followed by 30 cycles at 95°C for 45 s, 56°C for 45 s, 72°C for 45 s, and a final extension at 72°C for 10 min. The validated libraries were subjected to sequencing on the Illumina HiSeq 2500 platform (BGI, Shenzhen, China) following the standard pipelines of Illumina, thereby generating 2 × 250 bp paired-end reads.

#### 2.2.3 Sequencing and Bioinformatics Analysis

Sequencing and bioinformatics analysis were conducted as reported previously (19). Clustering analysis was conducted using QIIME (version 1.8.0) based on UPGMA. KEGG functional predictions were generated using the PICRUSt software. Bar plots and operational taxonomic unit (OTU) accumulation curves were plotted using the R package version 3.4.1 and version 3.1.1, respectively. Partial least-squares discrimination analysis (PLS-DA) was performed using the R package mixOmics. OTU rank curves were plotted using the R package version 3.1.1. A GraPhlan map of the species composition was generated using GraPhlAn. Significant genera or functions were determined using the Wilcoxon test or Kruskal test in R (version 3.4.1).

### 2.3 Untargeted Metabolomics Analysis of Serum

Metabolite extraction and liquid chromatography-mass spectrometry (LC-MS) were performed at Shanghai Applied Protein Technology following a previously reported protocol (20).

#### 2.3.1 Metabolite Extractions

Briefly, peripheral blood samples were collected in the morning before breakfast from the elbow of the participant using a disposable vacuum blood collector. The samples were then centrifuged at 3,000 × *g* for 10 min, and the supernatants were transferred to clean tubes. Then, 400 μL of cold extraction solvent methanol/acetonitrile/water (2:2:1, v/v/v) was added to 100 mg of serum sample and adequately vortexed to extract metabolites from serum samples. After vortexing, the samples were incubated on ice for 20 min and then centrifuged at 14,000 × *g* for 20 min at 4°C. The supernatant was collected and dried in a vacuum centrifuge at 4°C. The samples were re-dissolved in 100 μL of acetonitrile/water (1:1, v/v) solvent and transferred to LC vials for LC-MS analysis.

#### 2.3.2 LC-MS Analysis

Metabolite extracts were analyzed using a quadrupole time-of-flight MS (Sciex TripleTOF 6600) coupled to hydrophilic interaction chromatography *via* electrospray ionization for untargeted metabolomics of polar metabolites. LC separation was performed on an ACQUIY UPLC BEH Amide column (2.1 mm × 100 mm, 1.7 μm particle size) (Waters, Ireland) using a gradient of solvent A (25 mM ammonium acetate and 25 mM ammonium hydroxide in water) and solvent B (acetonitrile). MS was operated in both negative ion and positive ionization mode. The ESI source conditions were set as follows: Ion Source Gas1 as 60, Ion Source Gas 2 as 60, curtain gas as 30, source temperature of 600°C, IonSpray Voltage Floating (ISVF) ± 5500 V. In auto MS/MS acquisition, the instrument was set to acquire over the m/z range of 25–1000 Da, and the accumulation time for product ion scan was set at 0.05 s/spectra. The product ion scan was acquired using information-dependent acquisition (IDA) with high sensitivity mode.

#### 2.3.3 Data Analysis

Data analysis was performed as previously reported (21). After the data was preprocessed using Pareto-scaling, multidimensional statistical analysis, orthogonal partial least-squares discriminant analysis (OPLS-DA) was performed. The first principal component of the variable importance in the projection (VIP) was obtained from OPLS-DA to refine this analysis. The fold-change value of each metabolite was calculated by comparing the mean value between HCs and POI, POI and HRT, and HCs and HRT. The differential metabolites were further identified and validated using three databases, including Kyoto Encyclopedia of Genes and Genomes (KEGG)^1^, Human Metabolome Database, and Bovine Metabolome Database. The KEGG library was applied to the enrichment analysis of the KEGG metabolic pathway based on the differential metabolites. Hierarchical clustering was also performed as previously reported (22).

### 2.4 Detection of Serum Sex Hormones

Serum levels of FSH, E2, and progesterone were assessed using a competitive immunoassay. Peripheral blood samples were centrifuged at 3,000 × *g* for 10 min, and the supernatants were transferred to clean tubes. The assay was conducted using a Beckman Coulter Dx l800 system (174399; Beckman Coulter) strictly according to the manufacturer’s instructions.

### 2.5 Enzyme-Linked Immunosorbent Assay (ELISA) of Serum Cytokines

The blood samples were centrifuged at 3,000 rpm for 15 min at 4°C and stored at –80°C for subsequent serum determinations. The levels of IFN-γ (88-7316; Invitrogen), IL-6 (88-7066; Invitrogen), IL-17a (88-7117; Invitrogen), TGF-β1 (88-50390; Affymetrix eBioscience), IL-1β (1110122; Dakewe Biotech, Shenzhen, China), and TNF-α (1117202; Dakewe Biotech) were determined using the indicated ELISA kits following manufacturer’s instructions.

### 2.6 Animal Experiments

Female C57BL/6 (8-weeks old) mice were purchased from the Hangzhou Ziyuan Laboratory Animal Technology Co. Ltd. All mice were maintained under specific-pathogen-free conditions. All experimental procedures involving animals were conducted in accordance with the Guide for the Care and Use of Laboratory Animals (China), and the protocols were approved by the Animal Research Ethics Committee of Sir Run Run Shaw Hospital of Zhejiang University. The mice were free to drink water containing antibiotics for four days before *E. lenta* transplantation. The *E. lenta* group was administrated *E. lenta* (ATCC 25559) suspension at a dose of 2 × 10^8^ colony-forming units per 0.2 ml suspended in sterile anaerobic phosphate-buffered saline (PBS) orally twice per week for three weeks. Oral gavage of the same dose of heat-killed *E. lenta* was administered as the control. Seven days after *E. lenta* transplantation, mice received an intraperitoneal injection of PBS or E2 (100 μg/kg) daily for two weeks. Then, the mice were anesthetized and euthanized. The ovaries were processed for immunohistochemistry staining with TGF-β1 (ab215715, Abcam), α-SMA (ab124964, Abcam), and Collagen I (ab34710, Abcam) following the manufacturer’s instructions.

### 2.7 Statistical Analysis

For the 16S rRNA gene sequencing analysis, the beta diversity was estimated using QIIME (v1.8.0) at the OTU level, and the *P*-value was calculated using the Kruskal-Wallis multi-group comparison test. The nonparametric factorial Kruskal-Wallis rank-sum test was used to identify significantly different genera among different groups. *P* < 0.05 was considered statistically significant.

For the untargeted metabolomics analysis, significance was determined using an unpaired Student’s *t*-test. VIP value > 1 and *P* < 0.05 were considered statistically significant. KEGG pathway annotation was performed as previously reported (23). Fisher’s Exact Test was used to analyze and calculate the significance level of the enrichment pathway.

Data on the serum hormone and cytokine levels were statistically analyzed using Prism 6.0 (GraphPad version 6). All data are presented as the mean ± SEM. The means of two groups were compared using the student’s *t*-test and those of multiple groups were compared using a one-way analysis of variance. *P* < 0.05 was considered significant. Spearman’s rank correlation was used to calculate the correlation between microbiota genera and metabolite levels, microbiota genera and serum hormone and cytokine level, metabolite levels, and serum hormone and cytokine levels.

## 3 Results

### 3.1 HRT Reverses Serum Hormone and Profibrotic Factor Levels in Patients With POI

**Figure 2:**
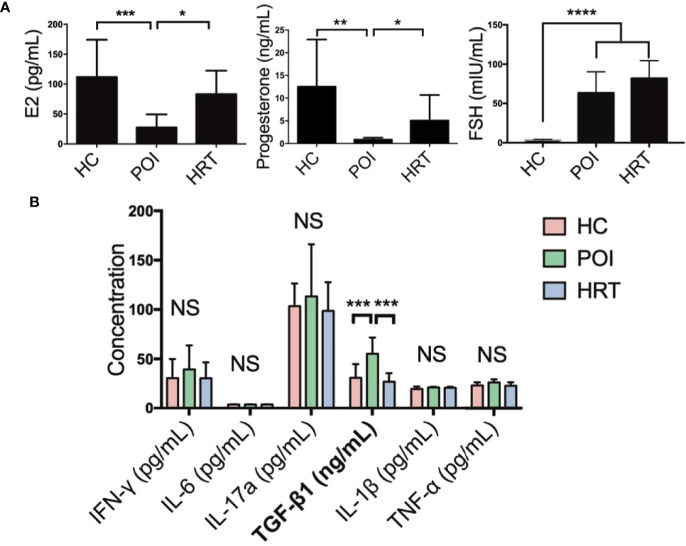
Serum hormone and cytokine levels in the three study groups. **(A)** Serum levels of E2, progesterone, and FSH in the three study groups. **(B)** Serum cytokine levels in the three study groups were compared using one-way analysis of variance with GraphPad Prism 6. Data are represented as mean ± SEM. n = 10 in each group, *P < 0.05, **P < 0.01, ***P < 0.001, ****P < 0.0001, NS, not significant.

We collected the serum samples from patients and detected the levels of serum sex hormones and cytokines (**Table 1**). Serum E2 and progesterone levels were significantly reduced in patients with POI compared to HCs, but these were reversed by HRT (**Figure 2A**). Serum FSH level was significantly increased in patients with POI compared to those in HCs; however, this increase was not reversed by HRT (**Figure 2A**). Serum levels (pg/mL) of the cytokines IFN-γ, IL-6, IL-17a, IL-1β, and TNF-α were not significantly affected by POI, except for TGF-β1 (ng/mL), whose level was significantly increased in patients with POI compared to that in HCs. This increase was reversed by HRT (**Figure 2B**). These data indicated that HRT may ameliorate the reduction in E2 and progesterone levels but does not affect high FSH levels in patients with POI and suppress fibrosis in patients with POI.

**Table 1:** Characteristics of the sample donors.

Parameters	HC (n=10)	POI (n=10)	HRT (n=10)	*P* Value		
Age (y)	32.70 ± 1.521	34.40 ± 1.551	33.40 ± 2.012	HC vs POI *P* =0.4439	POI vs HRT *P* =0.6985	HC vs HRT *P* =0.7845
BMI (kg/m^2^)	19.81 ± 0.474	21.06 ± 0.399	20.09 ± 0.561	HC vs POI *P* =0.0586	POI vs HRT *P* =0.1760	HC vs HRT *P* =0.7066
FSH (mIU/mL)	3.66 ± 0.195	63.45 ± 8.493	81.94 ± 7.142	HC vs POI *P*<0.0001	POI vs HRT *P* =0.1129	HC vs HRT *P*<0.0001
E2 (pg/mL)	111.70 ± 19.780	27.79 ± 6.767	83.08 ± 12.46	HC vs POI *P* =0.0008	POI vs HRT *P* =0.0010	HC vs HRT *P* =0.2365
P (ng/mL)	12.51 ± 3.298	0.859 ± 0.138	5.042 ± 1.784	HC vs POI *P* =0.0024	POI vs HRT *P* =0.0312	HC vs HRT *P* =0.0619

### 3.2 HRT Partially Attenuates Gut Microbial Dysbiosis in Patients With POI

**Figure 3:**
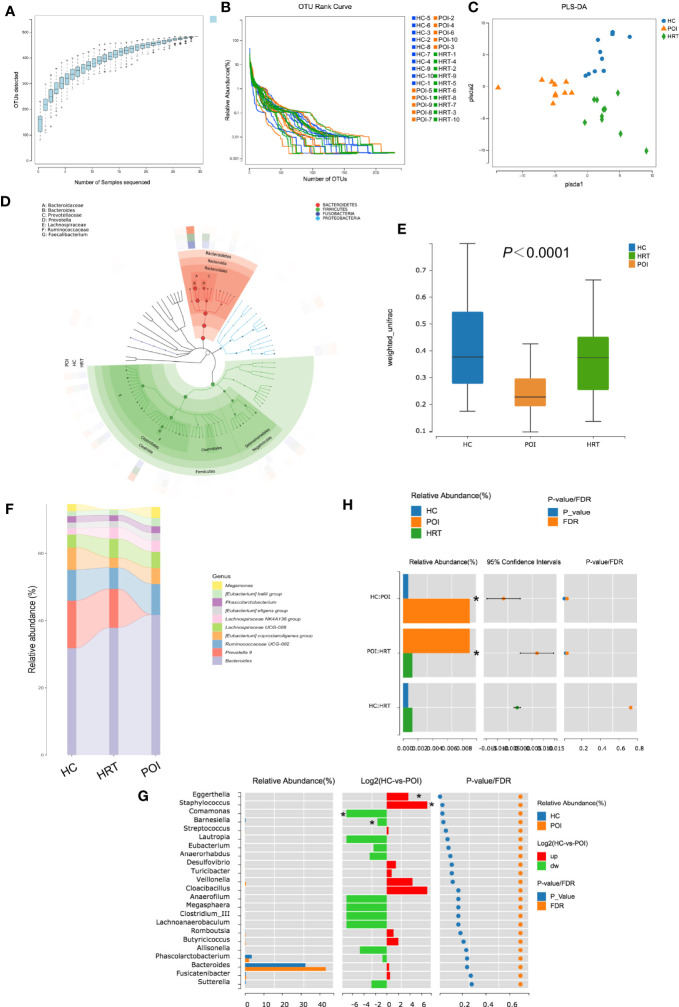
Alterations in gut microbial profiles in POI patients with or without HRT. **(A)** Operational taxonomic unit (OTU) accumulation curve. The horizontal coordinate represents the number of samples, and the vertical coordinate represents the number of OTUs (i.e., the number of genera detected). **(B)** OTU rank curve of each sample (blue: HCs; orange: POI patients; green: POI patients treated with HRT). The abscissa indicates the order of OTU abundance, and the ordinate indicates OTU abundance. **(C)** Partial least squares discriminant analysis (PLS-DA) of data obtained from the three groups showed grouped discrimination. **(D)** Evolutionary clade tree constructed based on the phylum, class, order, family, and genus (the 50 most abundant were selected). The clade tree was constructed from inside to outside, with each circle representing a level in the order phylum, class, order, family, and genus. Different phyla are colored differently, and the higher the number of nodes in the branching tree, the higher the abundance of the genus. The outer rings indicate abundance maps, and each ring represents a group of samples (one sample), and each group of samples corresponds to a color, the shade of which varies with the abundance of the genus. **(E)** Box plot of differences in beta diversity of the gut microbiota among the three study groups (n = 10/group). **(F)** Sankeyplot of highly abundant genera in the three study groups (sorted by the total mean abundance of each group). **(G)** Wilcoxon test of genus differences between healthy control and POI groups. A bar chart of the relative abundances in each group is shown on the left. The middle panel shows the log2 value of the average relative abundance ratio of the same species in the two groups. The panel on the right shows *p* values and false discovery rate values obtained by the Wilcoxon test, *P < 0.05. **(H)** Wilcoxon test of differences in the genus *Eggerthella* among the three groups, *P < 0.05.

As shown in **Figure 3A**, the OTU accumulation curve reflected the impact of the number of samples on genus diversity; flattening of the curve indicated a sufficient sampling quantity. The OTU richness of a sample is reflected by the length observed along the horizontal axis of the OTU rank curve; the wider the curve, the richer the OTU composition of the sample. The uniformity of OTUs in a sample is reflected by the shape of the curve along the vertical axis; the flatter the curve, the more uniform the species composition of the sample. No significant differences were observed in OTU richness and uniformity among the three study groups (**Figure 3B**). Furthermore, PLS-DA suggested that the overall gut microbiota composition differed among the three study groups (**Figure 3C**). The dominant phyla in the samples of the three groups were *Bacteroidetes*, *Firmicutes*, *Fusobacteria*, and *Proteobacteria* (**Figure 3D**). The β-diversity of the gut microbiota was decreased in patients with POI; this reduction was reversed by HRT (**Figure 3E**).

By analyzing the degree of similarity in bacterial taxonomy, we compared the abundance of gut microbiota at the genus level among the three groups. The top 10 abundant gut microbiota at the genus level among the three groups are shown in **Figure 3F**; however, no significant differences were observed at the genus level among the three groups (**Figure 3F**). The abundance of *Eggerthella* and *Staphylococcus* were increased, whereas that of *Comamonas* and *Barnesiella* was significantly decreased in patients with POI compared to that in HCs (*P* < 0.05) (**Figure 3G**). However, only the increase in the abundance of *Eggerthella* was significantly reversed by HRT (**Figure 3H**). These data indicated that the gut microbiota is dysbiotic in patients with POI, and HRT partially attenuates the gut microbiota dysbiosis in patients with POI.

### 3.3 HRT Reverses Serum Metabolite Alterations in Patients With POI

**Figure 4:**
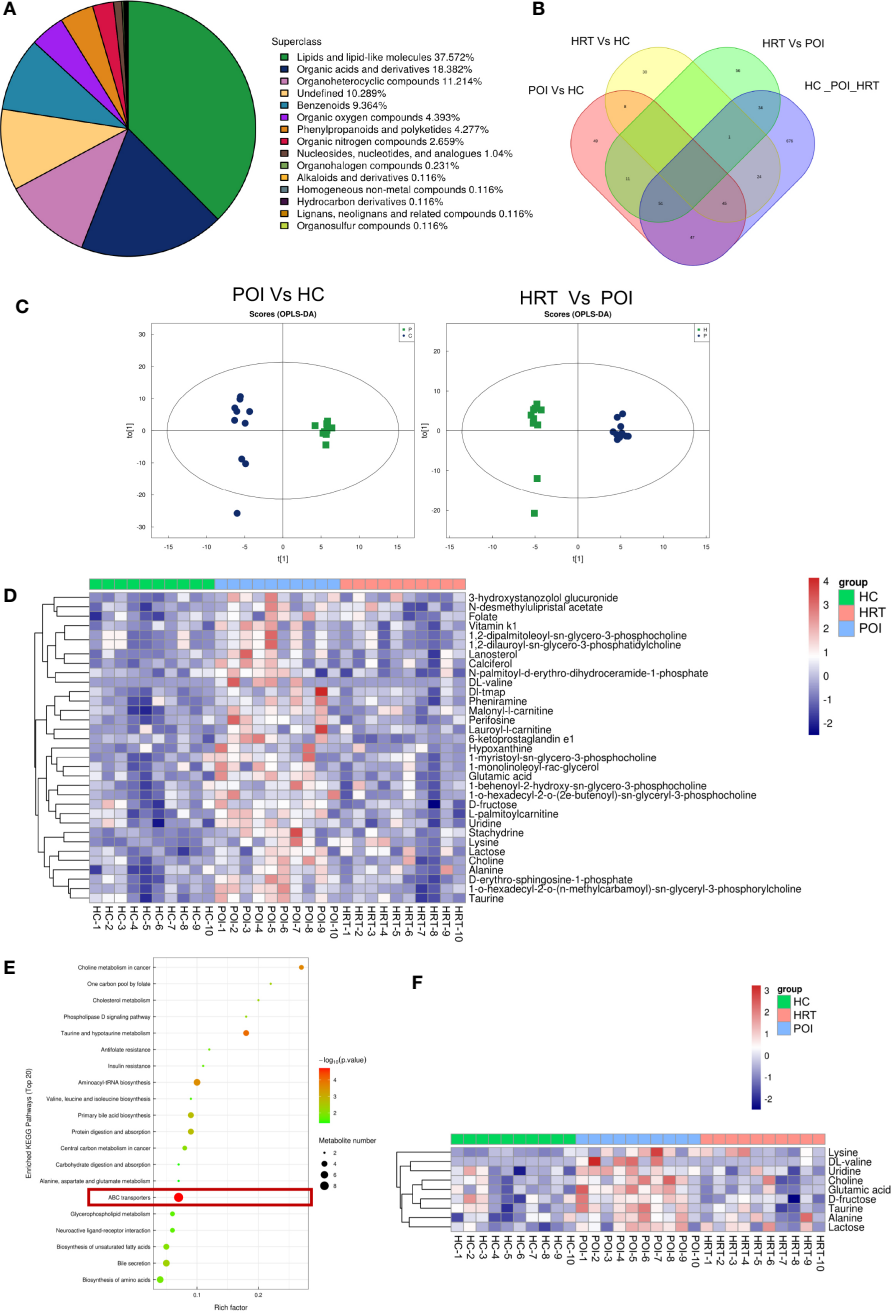
HRT reverses the metabolic alterations in the serum of patients with POI. **(A)** Subclass pie chart of the metabolites identified. **(B)** Venn diagram of metabolites identified in the sera obtained from the healthy controls, POI, and HRT groups. **(C)** Orthogonal prediction analysis of the underlying structure (by OPLS-DA) in positive mode to exclude the orthogonal variables irrelevant to the categorical variables in the metabolites. The horizontal coordinate represents the predicted score of the first principal component, and the vertical coordinate represents the score of the orthogonal principal component. **(D)** Heat map of hierarchical clustering analysis of the samples of the three groups. **(E)** Bubble diagram of KEGG enrichment analysis. **(F)** Heatmap of enriched metabolites involved in the pathway associated with ABC transporters.

To investigate potential associations between the gut microbiota and circulating metabolites in patients with POI, we profiled serum metabolites using a non-targeted metabolomics approach. Among all metabolites identified, lipids and lipid-like molecules were the most abundant, accounting for 37.572% of the total metabolites, followed by organic acids and derivatives, organoheterocyclic compounds, undefined compounds, benzenoids, organic oxygen compounds, phenylpropanoids and polyketides, and organic nitrogen compounds (**Figure 4A**). The distribution of the metabolites identified in the three groups is shown in a Venn diagram (**Figure 4B**). The supervised orthogonal projections to latent structures-discriminant analysis (OPLS-DA) score plots showed a clear separation between the HC and POI groups and between the POI and HRT groups (**Figure 4C**). The relative abundances of 33 significantly different metabolites (VIP value > 1 and *P* < 0.05) among the three groups, including 13 lipid and lipid-like molecules, nine organic acids and derivatives, two organoheterocyclic compounds, two organic oxygen compounds, two undefined compounds, and one organic nitrogen compound are shown in **Figure 4D**. The figure shows that the abundance of these circulating metabolites was substantially higher in patients with POI than that in the HC and HRT groups. These differential metabolites were further evaluated using the KEGG pathway analysis. The KEGG pathway analysis showed that the most enriched metabolites were ATP-binding cassette (ABC) transporters (**Figure 4E**) comprising nine metabolites (**Figure 4F**). The abundance of these nine metabolites was significantly increased in patients with POI. However, this increase was reversed by HRT (**Figure 4F**), suggesting that HRT reversed the alterations in serum metabolic profile in patients with POI.

### 3.4 Circulating Metabolites Are Correlated With the Gut Microbiota and Serum Factors

**Figure 5:**
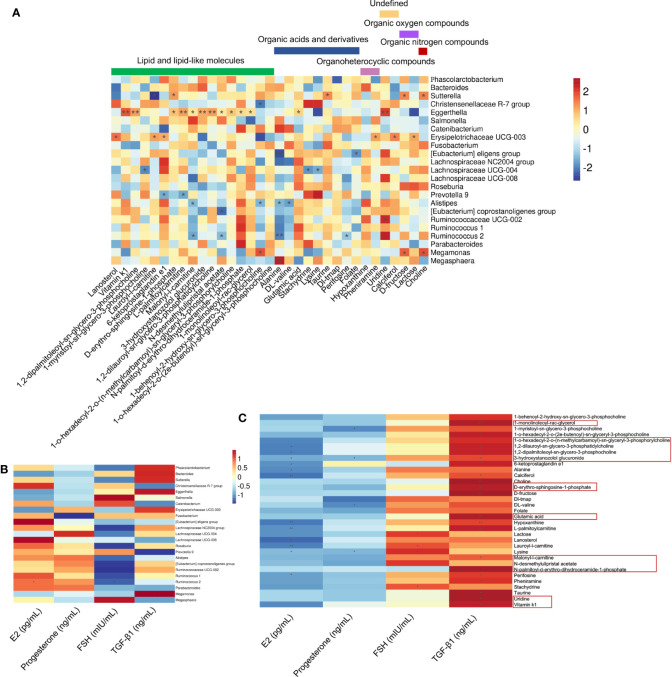
Associations between serum hormone and TGF-β1 levels, gut microbial species, and circulating metabolites. **(A)** Spearman’s rank correlations between 23 gut microbial species and 33 serum metabolites in healthy controls (n = 10), premature ovarian insufficiency (n = 10), and hormone replacement therapy (n = 10) groups. **(B)** Spearman’s rank correlations between 23 gut microbial species and serum estradiol (E2), progesterone, follicle-stimulating hormone (FSH), and TGF-β1 levels in the three study groups. **(C)** Spearman’s rank correlations between 33 serum metabolites and serum E2, progesterone, FSH, and TGF-β1 levels in the three study groups. Red indicates positive correlations between the bacterial genera and the metabolites, and blue indicates negative correlations between the bacterial genera and metabolites. *P<0.05, **P<0.01.

The correlations among 33 differential serum metabolites and 23 gut microbial genera among the three groups were analyzed (**Figure 5A**). We found that the abundance of *Eggerthella* was positively correlated with the serum levels of uridine, vitamin K1, 1,2-dipalmitoleoyl-sn-glycero-3-phosphocholine, L-palmitoylcarnitine, 3-hydroxystanozolol glucuronide, and 1,2-dilauroyl-sn-glycero-3-phosphatidylcholine (*P* < 0.01); and malonyl-l-carnitine, D-erythro-sphingosine-1-phosphate, N-desmethylulipristal acetate, 1-o-hexadecyl-2-o-(n-methylcarbamoyl)-sn-glyceryl-3-phosphorylcholine, N-palmitoyl-d-erythro-dihydroceramide-1-phosphate, 1-monolinoleoyl-rac-glycerol, and glutamic acid (*P* < 0.05) (**Figure 5A**). Among the metabolites that were significantly positively correlated with *Eggerthella*, except uridine, which was undefined, and glutamic acid, which is an organic acid, the other 10 metabolites were lipids and lipid-like molecules (**Figure 5A**). The abundance of *Eggerthella* was not significantly correlated with the serum levels of E2, progesterone, FSH, and TGF-β1 (**Figure 5B**).

Most of the 33 significantly different metabolites among the three groups were correlated positively with serum TGF-β1 levels and negatively correlated with serum E2 levels (**Figure 5C**). Among the metabolites that were significantly positively correlated with *Eggerthella*, 1-monolinoleoyl-rac-glycerol and glutamic acid were positively correlated with serum TGF-β1 levels (*P* < 0.01). Uridine, vitamin K1, malonyl-l-carnitine, D-erythro-sphingosine-1-phosphate, 1,2-dipalmitoleoyl-sn-glycero-3-phosphocholine, 1,2-dilauroyl-sn-glycero-3-phosphatidylcholine, and 3-hydroxystanozolol glucuronide were all positively correlated with serum TGF-β1 levels (*P* < 0.05), and the latter three metabolites were negatively correlated with serum E2 levels (*P* < 0.05) (**Figure 5C**). In addition, 6-ketoprostaglandin e1, choline, D-fructose, hypoxanthine, L-palmitoylcarnitine (*P* < 0.01), 1-o-hexadecyl-2-o-(n-methylcarbamoyl)-sn-glyceryl-3-phosphorylcholine, calciferol, perifosine, pheniramine, and taurine (*P* < 0.05) were positively correlated with serum TGF-β1 levels (**Figure 5C**).

### 3.5 Estrogen Ameliorated Ovarian Fibrosis Induced by *Eggerthella lenta* in Mice

**Figure 6:**
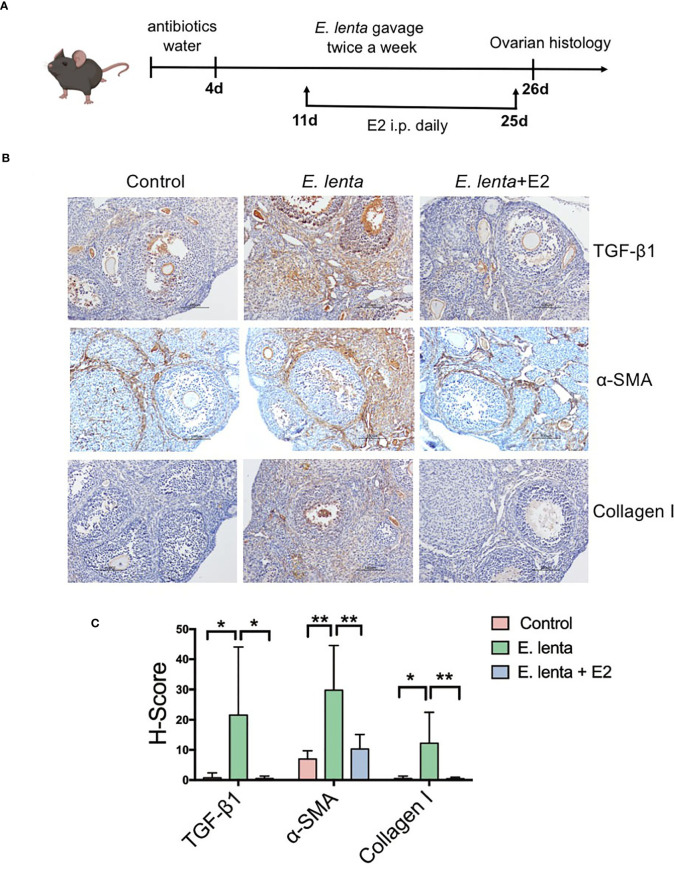
Estrogen ameliorated ovarian fibrosis induced by *Eggerthella lenta* in mice. **(A)** Schematic diagram of the animal experimental protocol. **(B)** The expression of TGF-β1, α-SMA, and collagen I in mice ovarian tissues was detected using immunohistochemistry. Data are representative images obtained upon examination of mice in the control, *E. lenta*, and *E. lenta* + E2 group. **(C)** Staining intensity of TGF-β1, α-SMA, and collagen I expression analyzed *via* H-SCORE analysis and compared using one-way analysis of variance (ANOVA), followed by Newman-Keuls multiple comparison test using GraphPad Prism 7 (n = 6); scale bar = 100 μm; mean ± SEM, (*P < 0.05, **P < 0.01, NS, not significant).

To further investigate the correlation between *Eggerthella* and ovarian fibrosis, mice were inoculated with *E. lenta*, the type species of genus *Eggerthella*, using oral gavage (**Figure 6A**). Compared with mice transplanted with sterile PBS, mice transplanted with *E. lenta* suspension demonstrated a significant increase in the expression of TGF-β1, α-SMA, and collagen I in the ovarian tissue, especially in the stroma and theca cells (**Figures 6B, C**), indicating increased levels of ovarian fibrosis. To confirm whether HRT ameliorated ovarian fibrosis induced by *E. lenta*, after inoculation with *E. lenta*, the mice were injected with or without E2 intraperitoneally daily. Compared with mice transplanted with *E. lenta* but without E2 supplementation, the expression of TGF-β1, α-SMA, and collagen I in the ovarian tissue reduced approximately to normal levels in the mice transplanted with *E. lenta* and supplemented with E2 (**Figures 6B, C**). These data reveal that estrogen ameliorated ovarian fibrosis induced by *E. lenta* in mice.

## 4 Discussion

This study demonstrated different abundances of *Eggerthella* in the gut and different serum levels of metabolites and TGF-β1 in patients with POI, and these alterations were reversed by HRT. Correlations among the altered factors were analyzed. The results demonstrated that serum metabolites, especially lipids and lipid-like molecules, were positively correlated with TGF-β1 levels and *Eggerthella*.

*Eggerthella* are anaerobic, nonsporulating, gram-positive bacteria of the family *Coriobacteriaceae*. This genus has been underrecognized for a long time, and their pathogenicity is poorly characterized (24). Vieira-Silva et al. (25) recently reported that *Eggerthella* may be associated with systemic inflammation. Abnormally-elevated abundance of *Eggerthella* induced the production of serum uremic toxins, including p-cresol sulfate, phenylacetylglycine, phenyl sulfate, indoxyl sulfate, creatinine, and urea, thereby aggravating renal fibrosis and promoting renal disease development (26). Increased abundance of *Eggerthella* has also been found in patients with sarcopenic cirrhosis and was correlated with the level of fibroblast growth factor 21 in cirrhosis patients (27). Autoimmune POI is characterized by ovarian fibrosis, which is caused by proinflammatory immune cell infiltration (28). The abundance of *Eggerthella* in the stool of patients with POI and its involvement in the development of POI remained unexamined in previous studies. This study revealed that the abundance of *Eggerthella* was increased in patients with POI, and this increase was reversed by HRT, suggesting that *Eggerthella* may be involved in the development of POI. Moreover, our findings indicate that HRT not only closely mimics the normal ovarian hormone production in patients with POI but also attenuates gut microbial dysbiosis, which is reportedly associated with fibrosis.

Among the 33 differentially expressed serum metabolites, *Eggerthella* abundance was primarily positively correlated with lipids and lipid-like molecules. The abundance of *Eggerthella* is increased in patients with Rett syndrome, and it induces alterations in short-chain fatty acids profiles (29). Colonization of *Eggerthella* in patients with coronary artery disease resulted in increased circulating cholesterol levels (30). The alteration of lipid metabolites in POI with respect to *Eggerthella* is unclear. Nevertheless, abnormalities in lipid metabolism and glucose metabolism and a reduction in kidney function have been reported in females with POI (31, 32). As mentioned earlier, an association between POI and increased risk of T2DM has also been recognized (7). These findings suggest that POI is not just a local lesion of the ovary but a systemic abnormality affecting multiple factors ranging from the gut microbiota to circulating lipid metabolites, which can be improved by HRT.

Different metabolites involved in the ABC transporter pathway were identified and enriched in the sera of patients with POI. In humans, ABC transporters represent the largest family of transmembrane proteins, which demonstrate crucial functions in cell homeostasis, including mitochondrial iron homeostasis, cholesterol metabolism, immunity, and drug response (33). Dysfunction of these transporters is reportedly associated with cystic fibrosis (33, 34). This study indicated that HRT may attenuate abnormalities in the blood metabolite profile of patients with POI, particularly abnormal levels of ABC transporters, which have been associated with cystic fibrosis.

TGF-β1 is a master regulator of extracellular matrix accumulation and, consequently, a potential key driver of fibrosis, which found to strongly promote fibronectin and collagen production (35–37). TGF-β1 is also a suppressor of excessive inflammation (38), however, we found that serum TGF-β1 was increased in POI patient without HRT, instead of decreased, and other inflammatory cytokines were not change statistically. An increased serum TGF-β1 level suggests a higher level of fibrosis in patients with POI; this increase is attenuated by HRT. We also found that the serum TGF-β1 level was positively correlated with circulating metabolites, which were also largely positively correlated with intestinal *Eggerthella*. These data suggest that HRT downregulates the expression of TGF-β1.

Nevertheless, this study has some limitations. The sample size of each group was too small. Although some intestinal genera and serum metabolites demonstrated changes with respect to abundance, these changes were not statistically significant. For example, the abundance of *Prevotella 9* was decreased and that of *Bacteroides* was increased in patients with POI; these increases were reversed by HRT (**Figure 3F**). However, these alterations were not statistically significant (P > 0.05), which may become significant after increasing the sample size. Larger sample size may help us discover more potential POI-related intestinal genera. Hence, further studies with larger sample sizes are needed in the future. In our study, animal experiments confirmed the correlation between intestinal *E. lenta* and ovarian fibrosis, but the serum metabolites were not detected simultaneously due to limitations in time and experimental conditions. The specific mechanism underlying the induction of ovarian fibrosis by *E. lenta* needs to be elucidated in the future.

In summary, our study revealed alterations in the gut microbiota, circulating metabolites, and serum cytokine profiles in patients with POI, and these alterations could be reversed by HRT. We also observed positive correlations between the gut microbiota and circulating metabolites and between circulating metabolites and TGF-β1 levels. Interactions between the gut microbiota, serum metabolites, and serum cytokines in patients with POI may play a critical role in the development of POI. This knowledge may pave the way for the development of preventive and curative therapies for patients with POI.

## Data Availability Statement

The datasets presented in this study can be found in online repositories. The names of the repository/repositories and accession number(s) can be found in the article/**Supplementary Material**.

## Ethics Statement

The studies involving human participants were reviewed and approved by Ethics committee of Sir Run Run Shaw Hospital, Zhejiang University School of Medicine. The patients/participants provided their written informed consent to participate in this study. The animal study was reviewed and approved by Animal Research Ethics Committee of Sir Run Run Shaw Hospital of Zhejiang University.

## Author Contributions

SZ, LJ, and JY supervised and designed the experiments. LJ, HF, JT, JZ, JJZ, YZ, XM, and HY collected and processed the clinical specimens. LJ and HF performed the experiments. XJ and ZS participated in discussing and revising the paper. LJ analyzed the data and wrote the paper. All authors contributed to the article and approved the submitted version.

## Funding

This work was supported by the National Key Research and Development Program of China (2018YFC1004800), the Key Projects Jointly Constructed by the Ministry and the Province of Zhejiang Medical and Health Science and Technology Project (WKJ-ZJ-2005), the Nature Science Foundation from the National Nature Science Foundation of China (NSFC) (81802336), the Nature Science Foundation of Zhejiang Province (LQ18H160005), and the Zhejiang Provincial Health Commission Innovative Talent Project (2020383552).

## Conflict of Interest

The authors declare that the research was conducted in the absence of any commercial or financial relationships that could be construed as a potential conflict of interest.

## Publisher’s Note

All claims expressed in this article are solely those of the authors and do not necessarily represent those of their affiliated organizations, or those of the publisher, the editors and the reviewers. Any product that may be evaluated in this article, or claim that may be made by its manufacturer, is not guaranteed or endorsed by the publisher.

## Footnotes

1
http://www.kegg.jp/
